# Development and Implementation of a COVID-19 Disease Response Protocol at a Large Academic Medical Center

**DOI:** 10.1017/dmp.2020.166

**Published:** 2020-05-22

**Authors:** Meshell Maxam, Kailynn J. DeRonde, Ana D. Vega, Dimitra Skiada, Christine A. Vu, Veronica Salazar, Renata Boatwright, Ennie Cano-Casillas, Venessa Goodnow, Kathleen A. Sposato, Peter G. Paige, David Zambrana, Don S. Steigman, Abdul M. Memon, Lilian M. Abbo

**Affiliations:** Department of Pharmacy Services, Jackson Memorial Hospital, Miami, Florida; Division of Infectious Diseases, Department of Medicine, Jackson Health System and University of Miami Miller School of Medicine, Miami, Florida; Department of Infection Prevention, Jackson Health System, Miami, Florida; Executive Office, Jackson Health System, Miami, Florida; Disaster and Emergency Preparedness Department, Jackson Health System, Miami, Florida

**Keywords:** COVID-19, development, implementation, protocol, SARS-CoV-2

## Abstract

In response to the rapid spread of novel coronavirus disease 2019 (COVID-19), health-care systems should establish procedures for early recognition and management of suspected or confirmed cases. We describe the various steps taken for the development, implementation, and dissemination of the interdisciplinary COVID-19 protocol at Jackson Health System (JHS), a complex tertiary academic health system in Miami, Florida. Recognizing the dynamic nature of COVID-19, the protocol addresses the potential investigational treatment options and considerations for special populations. The protocol also includes infection prevention and control measures and routine care for suspected or proven COVID-19 patients.

From the first report of novel coronavirus disease 2019 (COVID-19) in Wuhan, Hubei Province, China, the virus has spread to over 200 countries/territories in approximately 4 months, as reported by the World Health Organization (WHO). As of May 3, 2020, the WHO Situation Report states there are now 3,349,786 confirmed cases (84,393 within China and 3,265,393 outside of China), with 4643 deaths in China and 233,985 deaths in other countries.^1^ Since January 21, 2020, the Centers for Disease Control and Prevention (CDC) has reported 1,122,486 cases within the United States from 55 reporting states and jurisdictions. Thus far, 65,735 deaths have been reported in the United States due to COVID-19.^2^

As of May 4, 2020, there have been 35,969 positive cases in Florida, and 13,092 in Miami-Dade County.^3^ Jackson Health System (JHS) operates as a safety-net health-care enterprise for Miami-Dade County, including 3 acute care hospitals, a pediatric hospital, a behavioral health hospital, a rehabilitation hospital, 2 long-term care facilities, 5 urgent care centers, several outpatient clinics, and health services for 3 of Miami-Dade County’s corrections facilities. JHS has more than 12,000 employees, more than 1000 affiliated University of Miami faculty physicians, and serves a diverse population of approximately 2.8 million residents, plus additional travelers.

At JHS, the clinical and administrative leadership recognized early on the necessity for a comprehensive COVID-19 protocol and response team that could standardize communication, education, and preventive process measures across the diverse and complex health system. Their efforts, along with the work and research of the JHS Antimicrobial Stewardship Team (AST) for the investigational treatment protocol, and the Infection Prevention team, led to the quick development and dissemination of a clinical protocol. This protocol provides ongoing direction for clinicians, health-care workers, and other staff who provide care for patients under investigation (PUI) or with confirmed COVID-19. Due to the dynamic nature of COVID-19, the protocol has evolved, incorporating frequent updates and guidance as more is learned about this pandemic. The document contains best practices and guidance for diagnosis, isolation, appropriate personal protective equipment for the care of COVID-19 patients, guidelines for transportation, diagnostic tests, investigational COVID-19 treatment options, a summary of ongoing clinical trials at our facilities, how to mitigate additional transmissions, and education for patients and families.

Additionally, the protocol describes employee health guidelines for the screening and management of employees possibly exposed to or ill with this viral infection. In this report, we aim to describe the steps taken in the development and implementation of the interdisciplinary COVID-19 protocol at JHS as well as help guide other centers that have to rapidly implement emergency preparedness during this pandemic or any future epidemic health-care crisis. The most recent version of our protocol is available.^4^

## CREATION OF THE CORE GROUP

The first step in the development of the JHS COVID-19 protocol was assembling a core group. The clinical lead was the Chief of Infection Prevention and Antimicrobial Stewardship, and was supported by the Senior Director of Infection Prevention and Control. The core group was chaired by the Chief Medical Officer for Disaster and Emergency Preparedness with the Chief Physician Executive providing leadership support. The core group works closely with the rest of the health system’s leadership, the Antimicrobial Stewardship and Infection Prevention teams, the Florida Department of Health in Miami-Dade County (DOH-Miami-Dade), the Miami-Dade County Healthcare Preparedness Coalition, and the Miami-Dade County Office of Emergency Management.

From the very onset, the JHS Chief Operating Officer arranged 2 meetings per day. The first meeting’s purpose was to plan the operational response to COVID-19 across the health system, and included core team leaders, such as the JHS Chief Executive Officer and Chief of Staff, JHS Chief Medical Officer, JHS Medical Director of Infection Prevention and Control, Hospital Epidemiologist, Hospital Senior Director of Infection Prevention and Control, Chief information Officer, Chief Financial Officer, Chief Medical Officer of Disaster and Emergency Preparedness, Chief Human Resource Officer, Director of Public Safety/Emergency Management, Senior Vice President of Ambulatory Services and Long-Term Care Facilities, Chief Procurement Officer, Executive Vice President of Operations, and the Chief Marketing Officer. The second meeting included more than 100 physician leaders from critical care, the emergency department, and hospitalist medicine as well as nursing leadership and directors of critical care areas, nurse educators and other personnel from administration, laboratory, finance, public safety, respiratory therapy, procurement/supply chain, and the emergency department. Both meetings created a forum to address questions, provide daily reports, and develop and manage surge plans. These meetings ultimately evolved into the health system’s Incident Command and the subsequent development of command centers at each hospital for daily operations. This framework provided a health system infrastructure for the planning and managing of COVID-19 cases, and prepared our health system for various scenarios and contingency plans.

## DEVELOPMENT OF THE PROTOCOL

The protocol was designed following the epidemiological and clinical information provided by the CDC and DOH-Miami-Dade.^5^ The workflow was structured to maximize the opportunities for early identification of patients at risk of COVID-19 starting at the point of entry to JHS. The protocol guides health-care providers through the different steps in the recognition, isolation, testing, and management of PUI and confirmed cases, including employees, while maintaining the safety of themselves and other health-care providers. Specific details of the protocol, such as laboratory testing and treatment, required more frequent updates as information evolved.

Initial testing was performed in collaboration with the local and state DOH following the CDC guidelines, and rapidly evolved as our testing capacity was expanded as more was learned about the infection. Other laboratories, including private and in-house, were used once the test was available and validated. The implementation of in-house laboratories increased the number of patients that could be tested and drastically improved turnaround time. Any operational challenges, such as product malfunction or influx of samples, were resolved by sending samples to previously used laboratories. This process provided an opportunity to offload the in-house lab without compromising rapid results for PUIs. Screening questions are now used to guide testing in the electronic medical record at order entry. The questions are based on patient risk and location, which determines the prioritization and workflow in the laboratory. Tests are expedited by tiers according to the urgency of the case and molecular testing platform. For example, results can be made available within 15 min or have a turn-around time of 12-24 h.

Before the initial dissemination of the protocol, AST reviewed the literature for possible investigational treatment options for COVID-19. A literature summary table was created to include the study design, methods, results, and conclusions. This table allowed AST to add new literature as it was published and provided a comprehensive tool to quickly synthesize data. To optimize our guidelines and protocol, international meetings were conducted with intensivists working in highly prevalent countries, such as Italy and China. The need for intensive care input quickly became evident, and subsequent protocol updates included critical care intensivists, hospitalists, transplant and COVID-19 infectious diseases (ID) specialists, and rheumatologists. As new data became available, including the National Institutes of Health updated recommendations for the management of COVID-19 patients on April 21, 2020, the aforementioned stakeholders were involved in determining which investigational treatment options and clinical trials were available for the health system’s patient population. Following this decision, a therapeutics clinical trial core multidisciplinary team and, in parallel, a clinical management team were developed. The clinical team has evolved into daily virtual rounds between the intensive care unit (ICU), pharmacists, and ID consult teams to co-manage the therapeutic decisions.

Eventually discharge considerations were needed, and additional stakeholders became involved. Hospitalists and ID physicians, directors of case management, social work and infection control, nursing educators and ID nurse practitioners, information technology specialists, as well as transitions of care and ID pharmacists created and updated discharge recommendations.

## DISSEMINATION OF PROTOCOL

To ensure ease of accessibility, the initial release was shared by email on a weekly basis, with subsequent updates shared every day. The daily email was sent to the health system’s roughly 14,000 employees and medical staff members with a link to the latest version of the protocol. On average, the JHS COVID-19 email updates were opened 10,900 times a day, with the protocol being clicked on approximately 100 times per day. The protocol was also readily available on various platforms, including the health system’s intranet portal and 3 mobile applications: JacksonBadgeBuddy.org, JacksonCOVID19.org, and JHSMiami.org/stewardship. To date, JacksonBadgeBuddy.org’s COVID-19 section has been viewed 14,056 times, with the protocol downloaded 3614 times. JacksonCOVID19.org has been viewed 17,871 times, with the protocol downloaded 696 times. Metrics for JHSMiami.org/stewardship were not available at the time of publication.

Based on feedback, a PowerPoint presentation was created and updated frequently to provide an abbreviated version of the most important updates. Aside from physically disseminating the protocol, ongoing virtual meetings were held with various stakeholders to improve processes. The electronic medical record was used for testing guidelines and appropriate use of resources. Additionally, an admission power plan for COVID-19 and an electronic order set were created and built to ensure all labs, procedures, imaging, and treatments were ordered appropriately to monitor adverse events and drug interactions. Special signage was created for dedicated elevators to transport patients and at all JHS hospitals’ entry points. A special patient room sign for droplet or airborne isolation with eye protection and contact precautions was also created for COVID-19 cases to standardize the appropriate use of PPE and identify cases in the dedicated units. The COVID-19 core group remains in constant communication to ensure the most up-to-date information is made available. Daily challenges include screening of new information from government agencies, creating a unified protocol for multiple points of entry into the health system, and maintaining an updated protocol with constantly evolving information.

Support and recommendations from the executive leadership, CDC, DOH-Miami-Dade, and team leaders with experience in emergency preparedness were crucial to the success of rapidly implementing the protocol in all relevant clinical areas. On February 28, 2020, Florida Governor Ron DeSantis issued a declaration of public health emergency for COVID-19, and our staff was adequately prepared to deploy our protocol.

## INVESTIGATIONAL TREATMENT REVIEW PROCESS

Optimal treatment for COVID-19 has yet to be elucidated as several drugs are currently under investigation. Before clinical trials began, AST, ID, and critical care intensivists used the treatment guidelines that were created based on previously published literature, as well as CDC and WHO recommendations.^6-15^ Additional considerations were addressed for special populations, including pediatrics, pregnant women, and immunocompromised hosts in the treatment guideline.^16-28^ As clinical trials became available, the same group reviewed the inclusion and exclusion criteria and identified trials to meet the needs of the local patient population. In addition to these trials, some investigational drugs were made available through the manufacturer in the form of expanded access or compassionate use. The Investigational Drug Service worked in collaboration with AST, critical care intensivists, and ID to submit applications to become a study site. Before enrollment, all trials and programs were required to be approved by the JHS Clinical Research Review Committee. Once approved, patients could be enrolled based on the study design and criteria.

## CONCLUSION

In response to COVID-19, JHS has promptly developed a comprehensive protocol for early recognition and management of COVID-19. We have learned that constant communication with our medical staff and health-care workers is fundamental in any crisis or pandemic. In addition, the continuous dissemination of the protocol provides easy access to the most up-to-date information to ensure the safety and quality of care of patients and health-care workers. The protocol is featured in virtual town halls, videos, emails, newsletters, and Web/mobile applications, which serves as a central repository of information across a large, complex health system. We hope this manuscript can aid other institutions in their mission to manage this quickly spreading infection and plan for other pandemics in the future.
